# Off-clamp Versus On-clamp Robot-assisted Partial Nephrectomy: A Systematic Review and Quantitative Synthesis by the European Association of Urology Young Academic Urologists Renal Cancer Study Group

**DOI:** 10.1016/j.euros.2023.10.001

**Published:** 2023-10-28

**Authors:** Nikita Shrivastava, Gopal Sharma, Puneet Ahluwalia, Gagan Gautam, Selcuk Erdem, Daniele Amparore, Michele Marchioni, Nicola Pavan, Laura Marandino, Eduard Roussel, Riccardo Campi, Riccardo Bertolo, European Association of Urology Young Academic Urologists Renal Cancer Study Group

**Affiliations:** a Department of Urology, DKS Superspeciality Hospital and Postgraduate Institute, Raipur, India; b Urologic Oncology and Robotic Surgery, Medanta The Medicity, Gurugram, India; c Urologic Oncology Division, Urology Department, Faculty of Medicine, Istanbul University, Istanbul, Turkey; d Division of Urology, Department of Oncology, School of Medicine, San Luigi Gonzaga Hospital, University of Turin, Orbassano, Italy; e Laboratory of Biostatistics, Department of Medical, Oral and Biotechnological Sciences, G. D’Annunzio Chieti-Pescara University, Chieti, Italy; f Department of Urology, SS Annunziata Hospital, G. D’Annunzio Chieti-Pescara University, Chieti, Italy; g Unit of Urology, Department of Surgical, Oncological and Oral Sciences, P. Giaccone University Hospital, Palermo, Italy; h Clinical Research Fellow in Renal & Melanoma, Royal Marsden Hospital, London, UK; i Department of Urology, University Hospitals of Leuven, Leuven, Belgium; j Unit of Urological Robotic Surgery and Renal Transplantation, Careggi Hospital, University of Florence, Florence, Italy; k Department of Experimental and Clinical Medicine, University of Florence, Florence, Italy; l Urology Unit, San Carlo di Nancy Hospital, Rome, Italy

**Keywords:** Off-clamp, Ischemia, Partial nephrectomy, Kidney cancer

## Abstract

**Context:**

The superiority of off-clamp robot-assisted partial nephrectomy (RAPN) over the on-clamp technique has recently been questioned by randomized controlled trials comparing the two techniques.

**Objective:**

To systematically review the recent literature and perform a quantitative synthesis of data on the comparison of off-clamp versus off-clamp hilar control during RAPN.

**Evidence acquisition:**

A systematic search was performed in the PubMed, Embase, Web of Science, and Scopus databases for studies comparing off-clamp versus on-clamp RAPN in terms of perioperative and functional outcomes. The study protocol was registered in the PROSPERO database (CRD42023413160). Only prospective randomized controlled trials and retrospective matched observational studies were included. The primary outcome of the study was the percentage decrease in the estimated glomerular filtration rate (eGFR).

**Evidence synthesis:**

A total of 11 studies were included involving a total of 2483 patients (944 patients in the off-clamp and 1539 patients in the on-clamp group). There was no difference between the two groups in the percentage decline in eGFR (mean difference [MD] 0.04%, 95% confidence interval [CI] −3.7% to 3.86%; *p* = 0.98). There were so significant differences between the groups for length of hospital stay (*p* = 0.56), complications (*p* = 0.08), conversion to open or radical surgery (*p* = 0.18), estimated blood loss (*p* = 0.06), or need for blood transfusion (*p* = 0.07). The operative time was shorter in the off-clamp group (MD−21.89 min, 95% CI −42.5 to −1.27; *p* = 0.04) but after sensitivity analysis the difference was no longer statistically significant (*p* = 0.15). The positive surgical margin rate was significantly lower in the off-clamp group (odds ratio 0.6, 95% CI 0.39–0.91; *p* = 0.02).

**Conclusions:**

Our review revealed no clinically relevant differences in perioperative and functional outcomes between off-clamp and on-clamp RAPN.

**Patient summary:**

In this review, we compared the two methods of controlling the kidney blood vessels during robot-assisted surgery to remove part of the kidney. We noted that there was no difference between the two groups for outcomes such as complications and the decrease in kidney function after surgery.

## Take Home Message

Comparison of off-clamp versus on-clamp robotic partial nephrectomy revealed no difference between the groups in percentage decline in estimated glomerular filtration rate at last available follow-up, operative time (sensitivity analysis), complication rates, need for blood transfusion, estimated blood loss, or conversion to open or radical surgery. The positive surgical margin rate was lower in the off-clamp group.

## 1 Introduction

Partial nephrectomy (PN) has become the standard of care for management of the majority of localized renal masses [1]. The preservation of renal function and the reduction in the risk of surgically induced chronic kidney disease are the main advantages of PN over radical nephrectomy [1]. PN performed using a robotic platform (robot-assisted laparoscopic PN, RAPN) has become the preferred approach whenever the technology is available [1]. To reduce blood loss and facilitate tumor excision during surgery, the renal vessels have typically been clamped during RAPN. The resulting warm ischemia has historically been considered a risk factor for postoperative renal function because of ischemic injury. Initial studies on the topic have shown that the duration of the ischemia is an important predictor of postoperative outcomes and preserved renal function [2], [3].

Several prospective or retrospective observational studies and two prospective randomized controlled trials (RCTs) have compared off-clamp versus on-clamp RAPN [4], [5], [6], [7]. Both RCTs showed no benefit on postoperative renal functional outcomes with renal artery clamping, whereas the findings from observational studies are been contradictory [4], [5], [8]. The issue has also been addressed in at least three systematic reviews [9], [10], [11]. A review by Antonelli et al. [9] revealed no difference between the two techniques in terms of postoperative renal function. Another by Deng et al. [10] showed no difference in terms of long-term renal function preservation. However, the short-term estimated glomerular filtration rate (eGFR) and serum creatinine levels favored the off-clamp group [10]. Lastly, Huang et al. [11] noted in their review that the off-clamp group had better postoperative renal function preservation.

These previous reviews were not without limitations. The study by Deng et al. [10] was heterogeneous as it included studies on robotic as well as laparoscopic PN. The other two reviews did not make an attempt to ensure comparable baseline characteristics between the two groups. Therefore, considering the limitations noted in the previous reviews, we aimed to include only RCTs and propensity-matched studies comparing off-clamp versus on-clamp RAPN in patients with renal masses.

## 2 Evidence synthesis

### 2.1 Study design

Our aim was to perform a systematic literature search for studies comparing on-clamp versus off-clamp RAPN. Before the start of the systematic review, the protocol was specified and registered in the PROSPERO database (CRD42023413160). During the conduct of this study, the PRISMA (Preferred Reporting Items for Systematic Reviews and Meta-Analyses) guidelines [12] and the Cochrane handbook version 5.1.0 [13] were followed.

### 2.2 Search strategy

A literature search was performed by two study authors independently using the PubMed/MEDLINE, Embase, Scopus, and Web of Science databases. The literature was searched from databases inception up to May 2, 2023. Filters as for language (“English”) and subject (“Human”) were applied. Additional articles were sought from the reference lists in the reviews previously published on the topic and the articles that were selected for full-text review. We did not consider conference abstracts for inclusion in the review.

We followed the PICO (Population, Intervention, Comparator, Outcome) methodology to design our search strategy. The population was patients who underwent RAPN. The intervention was the off-clamp (also known as clampless) approach to the renal hilum. The comparator was patients for whom dissection and clamping of the renal artery were included as part of the surgery (on-clamp). The primary outcome was the postoperative change in renal function. Both keywords and MeSH terms were used to develop a search strategy.

The search string used for the review was as follows: ((robot-assisted partial nephrectomy) AND ((on-clamp) OR (on clamp))) AND (((off-clamp) OR (off clamp)) OR (clampless)).

Two authors (G.S. and N.S.) initially screened titles and abstracts for inclusion in the study after removing duplicates. In cases of discrepancy, the help of a senior author was sought (G.G.). The search strategy for PubMed is provided in Supplementary Table 1.

### 2.3 Inclusion criteria

Prospective randomized controlled trials comparing on-clamp and off-clamp RAPN were included. Prospective or retrospective nonrandomized cohort studies were eligible for inclusion only if matching for baseline characteristics was performed.

### 2.4 Exclusion criteria

We excluded studies describing either of the techniques alone or in combination with other techniques. Case reports, case series, conference abstracts, reviews, and letter were also excluded.

### 2.5 Outcomes

The primary aim was to compare the percentage decrease in estimated glomerular filtrate rate (eGFR) between the off-clamp and on-clamp RAPN groups at last follow-up.

Secondary aims were to compare other perioperative outcomes between the groups, including operative time, length of stay (LOS), complication rate, estimated blood loss (EBL), positive surgical margin (PSM) rate, need for blood transfusion, and conversion to open or radical nephrectomy.

### 2.6 Data extraction

Data extraction was performed independently by the two review authors using a unique predefined data template. Data were checked for consistency. The predefined data template included first author name, country, year of publication, number of patients, mean age, sex, RENAL nephrometry score (RNS), preoperative creatinine, preoperative eGFR, LOS, EBL, PSM rate, complications, need for blood transfusion, and conversion to open or radical nephrectomy. For studies in which propensity matching was performed, post-matching data were extracted and considered for analysis.

### 2.7 Risk of bias

For RCTs and cohort studies, the Cochrane risk of bias (RoB) assessment tool [14] and Newcastle Ottawa scale (NOS) [15] were used to assess the quality of studies. In accordance with PRISMA recommendations, two review authors (G.S. and N.S.) assessed the study quality and discrepancies were resolved by consulting a senior author (G.G.).

### 2.8 Statistical analysis

Statistical heterogeneity was examined using χ^2^ and I^2^ tests. A *p* value of <0.10 was considered to signify the presence of significant statistical heterogeneity. In the presence of heterogeneity, a random-effects model was used. Otherwise, a fixed-effect model was used. For categorical variables, the Mantel-Haenszel method with odds ratio (OR) as the effect measure was used for pooling data in the meta-analysis. The inverse variance (IV) method with mean difference (MD) as the effect measure was used for continuous variables. The mean and standard deviation (SD) were required for pooling of data for continuous variables. The mean and SD were estimated from the median and range or interquartile range (IQR). Visual examination of a funnel plot for the primary outcome was used to determine publication bias. A symmetric curve denotes an absence of publication bias and an asymmetric curve the presence of publication bias. Analysis was performed using RevMan version 5.4 software (Cochrane Collaboration, Copenhagen, Denmark).

## 3 Evidence synthesis

### 3.1 Search strategy and study selection

**Fig. 1:**
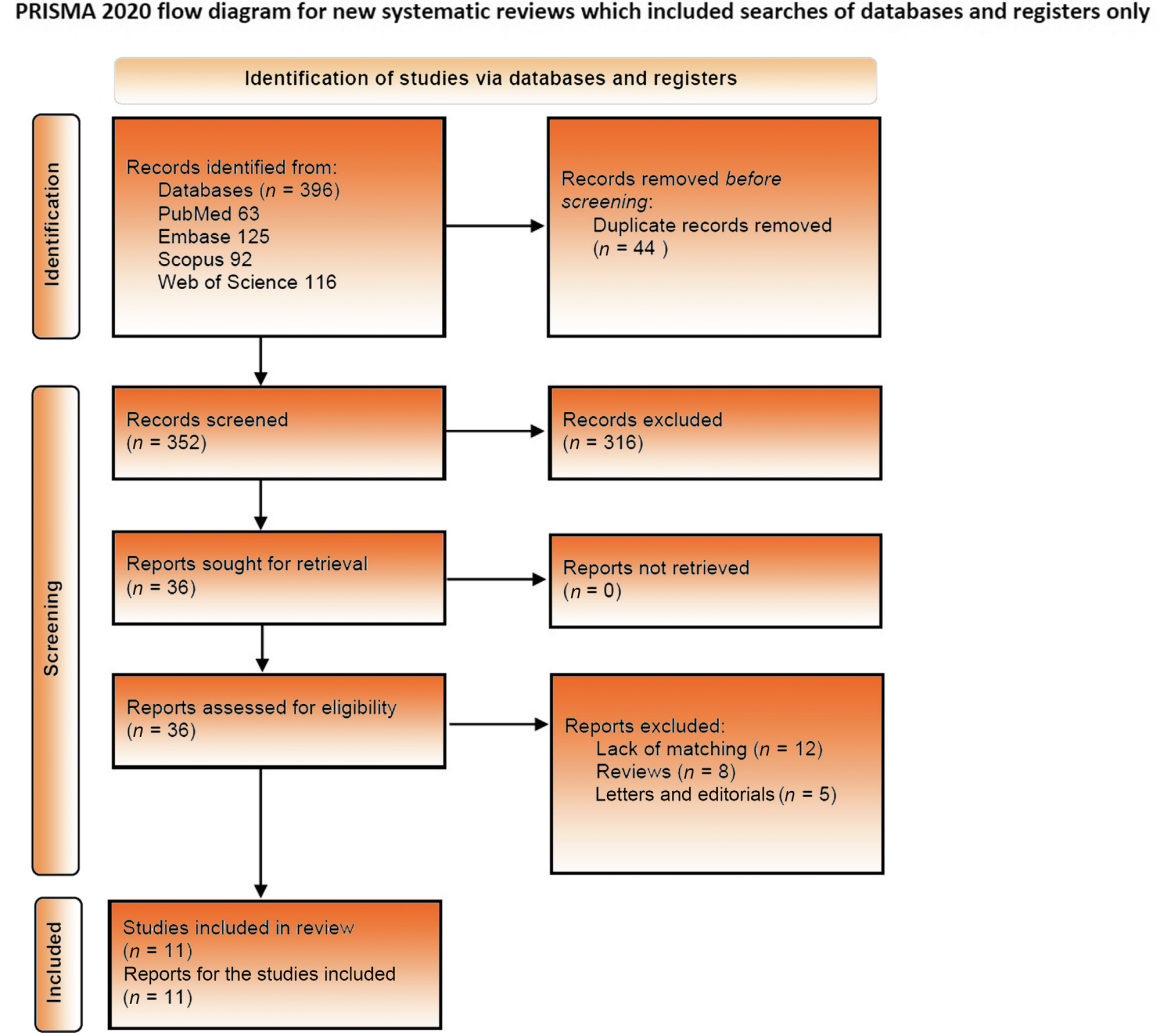
PRISMA flow chart showing the process for identification, screening, and inclusion of studies.

The literature search yielded a total of 396 articles (PubMed 63, Embase 125, Scopus 92, and Web of Science 116). References were imported via a citation manager and duplicates were removed (*n* = 44). After title and abstract screening, 36 articles were selected for full-text review. Of these, 11 studies were eligible for inclusion in the pooled analysis after checking against the inclusion criteria [5], [6], [7], [8], [15], [16], [17], [18], [19], [20], [21] (Fig. 1).

### 3.2 Study characteristics

Among the studies included, two were RCTs and nine were matched cohort studies (prospective or retrospective) involving a total of 2483 patients. Of these, 944 patients had undergone off-clamp RAPN and the remaining 1539 underwent on-clamp RAPN. Most of the studies included were multicenter studies with 1:1 matching. Among the cohort studies, the variables used for matching were heterogeneous (Table 1).

**Table 1:** Characteristics of the studies included in the review

Study, year, and country	Multicenter	Design	Patients (off-C/on-C)	Variables matched	Matching ratio	NOS score
Anderson [15], 2017, USA	No	PS	50/50	Age, preoperative renal function, tumor size, and RNS	1:1	7
Bertolo [20], 2018, Italy	Two-center	PS	200/400	Age, sex, smoking status, diabetes, hypertension, ASA score, solitary kidney status, preoperative eGFR, clinical tumor size, and RNS	2:1	7
Brassetti [6], 2022, Italy	Yes	RS	89/89	age, ASA score, eGFR at baseline, RENAL score	1:1	7
Kaczmarek [7], 2013, USA	Yes	RS	49/283	Surgeon’s experience, age, sex, race, BMI, ASA score, RNS, and eGFR	4:1	8
Mari [16], 2018, Italy	No	RS	120/120	Tumor side, polar tumor location, clinical T stage, calyceal system, and sinus compression/invasion	1:1	8
Peyronnet [19], 2017, France	Yes	RS	26/104	RNS, tumor size, and surgeon’s experience	4:1	9
Rosen [17], 2017, USA	Yes	RS	41/82	Age, sex, BMI, ASA score ≥3, diabetes, hypertension, coronary artery disease, baseline eGFR, tumor size, RNS, tumor laterality, lateral vs anterior/posterior tumor location, endophytic percentage, and operating surgeon	2:1	7
Tanagho [18]^,^ 2012, USA	No	PS	29/29	Not specified	1:1	7
Sharma [21], 2023, India	Yes	PS	205/205	Age, sex, BMI, RNS, and preoperative eGFR	1:1	8
Anderson [5], 2018, USA	No	RCT	40/40	–	–	–
Antonelli [8], 2019, Italy	Yes	RCT	95/137 (per protocol)	–	–	–

ASA = American Society of Anesthesiologists; BMI = body mass index; off-C = off-clamp; on-C = on-clamp; RNS = renal nephrometry score; eGFR = estimated glomerular filtration rate; NOS = Newcastle-Ottawa Scale; PS = prospective study; RS = retrospective study; RCT = randomized controlled trial.

### 3.3 RoB assessment

**Fig. 2:**
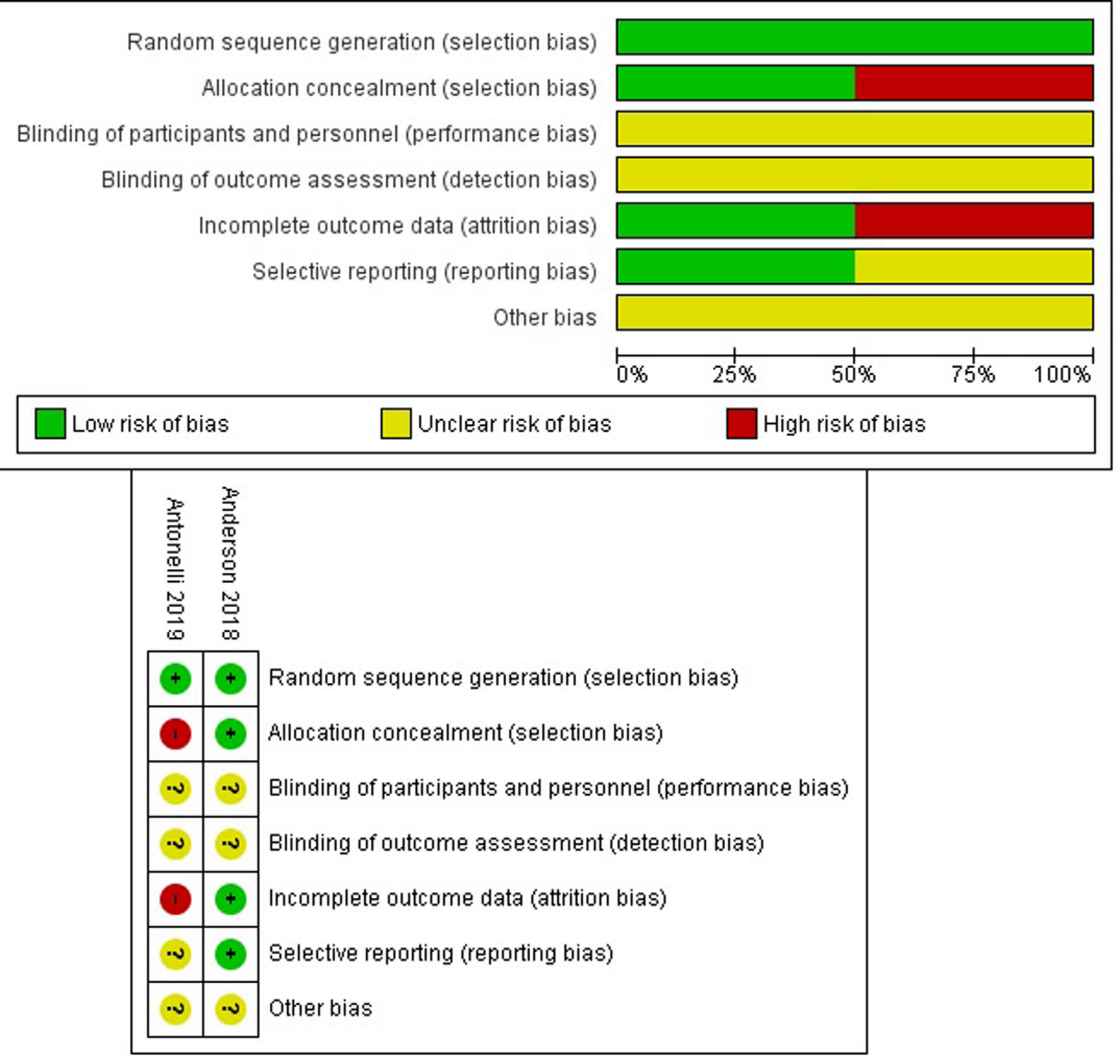
Risk-of-bias assessment for the randomized studies included in the review.

Quality assessment of the cohort studies revealed an NOS score ranging from 7 to 8 for the studies included (good quality). RoB assessment for the two RCTs is shown in Figure 2. Specifically, the study by Anderson et al. [5] had clear description of the randomization and allocation techniques and therefore was at low RoB for these variables. The study by Antonelli et al. [8] had clearly defined random sequence generation but not allocation concealment. Both studies were at unclear RoB for performance and detection bias.

### 3.4 Primary outcome

**Fig. 3:**
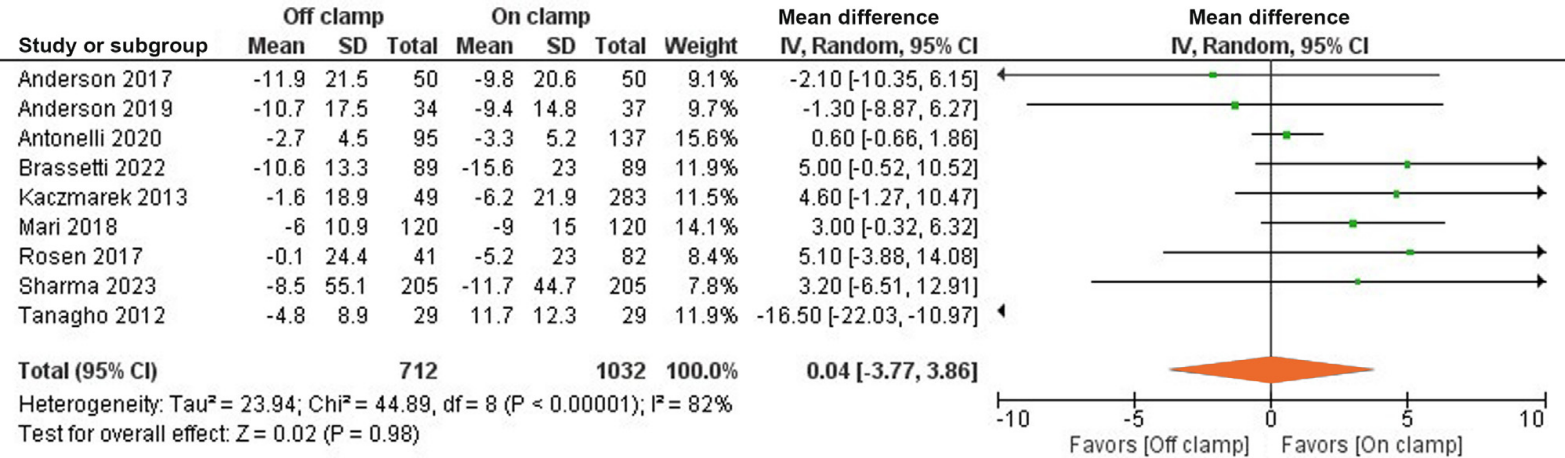
Forest plot for analysis of the primary outcome of the percentage decline in the estimated glomerular filtration rate.CI = confidence interval; df = degrees of freedom; IV = inverse variance; SD = standard deviation.

Data for the eGFR decline at last follow-up were extracted from nine studies involving 1744 patients. There was no difference between the groups for the percentage decline in eGFR (MD 0.04%, 95% CI −3.77% to 3.86%; *p* = 0.98). Random-effect IV analysis was used in view of significantly high heterogeneity, with I^2^ = 82% and χ^2^ = 44.8 (Fig. 3).

### 3.5 Secondary outcomes

**Fig. 4:**
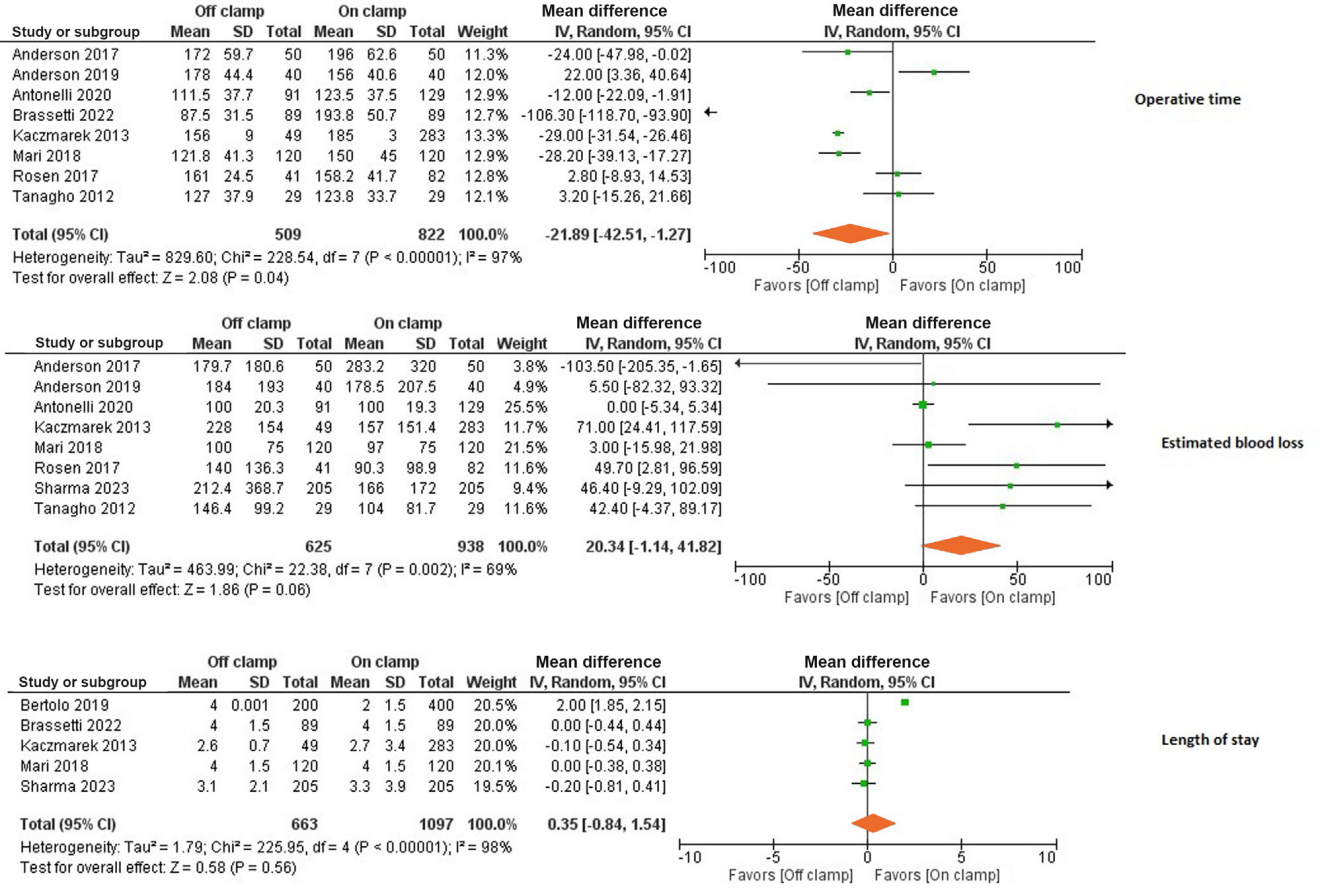
Forest plot of analyses for the operative time, estimated blood loss, and length of stay.CI = confidence interval; df = degrees of freedom; IV = inverse variance; SD = standard deviation.

Data on operative time were extracted from eight studies involving 1331 patients. On IV random-effect analysis, operative time was significantly shorter in the off-clamp group (MD −21.9 min, 95%CI −42.5 to −1.27; *p* = 0.04; Fig. 4).

**Fig. 5:**
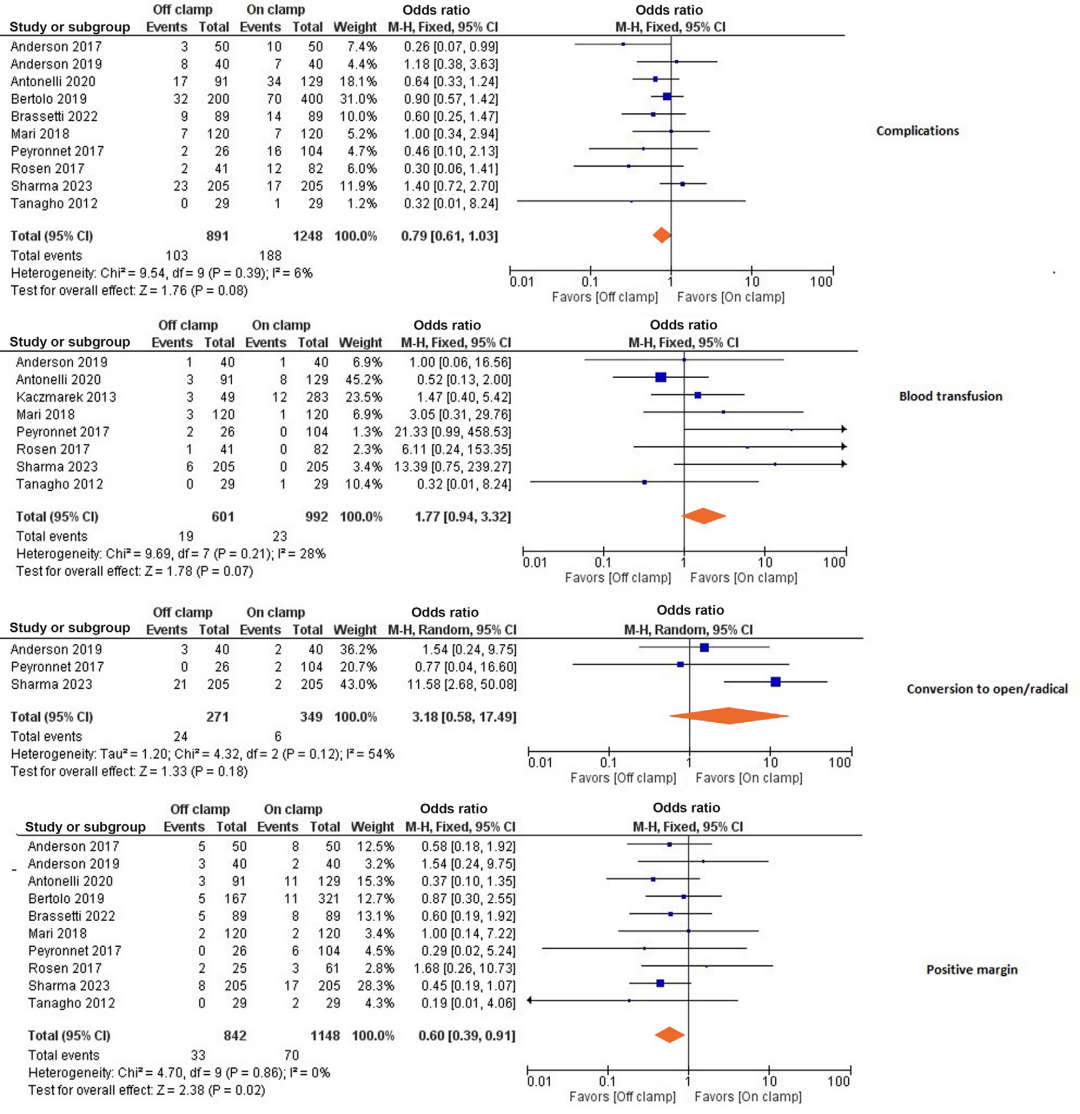
Forest plot of analyses for complications, blood transfusion, conversion to open or radical nephrectomy, and the positive surgical margin rate.CI = confidence interval; df = degrees of freedom; M-H = Mantel-Haenszel.

EBL during surgery did not significantly differ between the two groups (MD 20.34 ml, 95% CI −1.14 to 41.8; *p* = 0.06). Furthermore, there was no difference between the groups in LOS (MD 0.35 d, 95% CI −0.84 to 1.54; *p* = 0.56). Similarly, there was no significant difference between the groups for complications (OR 0.79, 95% CI 0.61–1.03; *p* = 0.08), conversion to open or radical nephrectomy (OR 3.18, 95% CI 0.58–17.5; *p* = 0.18), or need for blood transfusion (OR 1.77, 95% CI 0.94–3.32; *p* = 0.07; Fig. 4, Fig. 5).

The PSM rate was significantly lower in the off-clamp group (OR 0.60, 95% CI 0.39–0.91; *p* = 0.02; Fig. 5).

### 3.6 Sensitivity analysis and publication bias

Sensitivity analysis was performed for all the outcomes including the primary outcome. We excluded each study one at a time from the analysis and still noted no impact on the outcome parameters except for operative time. Analysis of the operative time appeared to be influenced by just the study by Brassetti et al. [6]. After excluding this study from the analysis, there was no significant difference between the two groups for operative time (MD 3.20 min, 95% CI −23.7 to 3.6; *p* = 0.15). Visual inspection of a funnel plot for the primary outcome (eGFR decline at last follow-up) showed a symmetric plot suggestive of no publication bias (Supplementary Fig. 1).

### 3.7 Discussion

Preservation of renal function is one of the main advantages of PN over radical nephrectomy [1]. Beyond unmodifiable patient and disease factors, there are several intraoperative technical steps for optimizing postoperative renal function, including the resection technique, the renorrhaphy technique, and management of the renal pedicle [22], [23]. Off-clamp RAPN is a modification of the “conventional” technique, whereby it was postulated that omission of clamping of the renal vessels would result in better preservation of postoperative renal function. However, avoidance of renal artery clamping could increase the risk of intraoperative complications such as bleeding, and compromised tumor resection in particular, with a risk of PSMs. Initial small retrospective studies comparing off-clamp versus on-clamp PN for well-selected renal tumors showed better functional outcomes and comparable perioperative outcomes [24]. This fueled the enthusiasm for off-clamp RAPN in well-selected patients. However, with emerging evidence, the advantages of off-clamp over on-clamp RAPN have become blurred. Despite the evidence from RCTs conducted in the recent past on the nonsuperiority of the off-clamp technique over on-clamp RAPN, surgeon experience and preferences are predominant in deciding on the technique to use for hilar clamping during RAPN. Our aim was to generate further evidence through a comprehensive literature search for studies comparing these two approaches in RAPN.

Our analysis revealed no difference in percentage decline in eGFR at last follow-up between the two groups. Furthermore, results for operative time (post sensitivity analysis), complication rates, need for blood transfusion, EBL, and conversion to open or radical surgery were comparable between the groups. Although on-clamp surgery facilitates tumor resection via hilar control during RAPN, the PSM rate was significantly higher in this group. This could very well be attributed to selection bias that could not be controlled, even via matching for baseline variables. For instance, regarding tumor complexity, most of the studies matched the two groups for RNS. RNS in itself is not a comprehensive parameter for defining the degree of surgical complexity. RNS includes only tumor-related factors and omits patients-related factors such as perinephric fat and visceral obesity.

Our findings are quite different from those from previous meta-analyses on this topic. In a meta-analysis of nine observational studies involving 896 patients by Cacciamani et al. [25], the authors found that off-clamp RAPN was associated with shorter operative time, higher EBL, and superior preservation of renal function. However, the studies included in this review were of limited quality and suffered from selection bias, high heterogeneity, and lack of comparability of the two groups for baseline variables. The first RCT comparing on-clamp versus off-clamp RAPN was reported by Anderson et al. [5], with 40 patients in each group. The authors observed no difference between the two groups for EBL, complications, oncologic outcomes, and postoperative renal function. This RCT was small, inadequately powered, and reported short-term outcomes. The next review, by Antonelli et al. [9], included 15 studies (one RCT and 14 retrospective studies) involving 2075 patients. The authors concluded that the off-clamp group had lower EBL and a shorter operative time, but there were no significant differences in complications or oncologic and functional outcomes between the two groups. The authors acknowledged the lack of comparability of the two groups at baseline and attributed the results to the greater use of the off-clamp technique for small renal masses [9].

Antonelli et al. [8] reported the largest RCT on this topic, with 324 patients randomly allocated to off-clamp or on-clamp RAPN. The study had well-defined criteria for selecting the operating surgeon, the follow-up protocol, and the method for calculating renal function, which were major strengths of the study. However, a major drawback of the study was large crossover between the two groups at the surgeon’s discretion. This crossover contaminated the overall results. To ensure comparability of this study with the others included, we decided to use the study per-protocol analysis for our meta-analysis. Moreover, Antonelli et al. [8] did not observe any difference in renal function preservation between the two groups, even at 24-mo follow-up. Finally, in the latest and largest meta-analysis by Huang et al. [11] on this topic, involving 21 studies and 4493 patients, the authors found that off-clamp RAPN was associated with significantly better functional outcomes. However, this study suffered from similar limitations to previous reviews and lacked comparability for baseline variables. Therefore, the resulting selection bias and lack of control for confounding factors makes interpretation of the results difficult.

Our review included only RCTs and studies in which the groups were matched for baseline variables, and focused on patients undergoing RAPN. Despite these strengths, some limitations must be acknowledged. First, evidence from this review is largely drawn from retrospective or prospective observational studies. Therefore, despite our best efforts to screen and include studies only with propensity matching, the possibility of selection bias cannot be completely ruled out. Second, surgeon experience is an important confounding factor in balancing off-clamp and on-clamp groups. Very few studies matched the two groups for this variable. Third, there was heterogeneity in the size and complexity of tumors among the studies. For instance, both RCTs only recruited patients with low- to moderate-complexity tumors and the study by Brassetti et al. [6] included only T2 tumors. Fourth, most of the studies reported eGFR as the primary measure of renal function. Limitations associated with eGFR calculation and variable methods for calculating eGFR among the studies could make results difficult to interpret. Furthermore, variability in timing of eGFR calculation across the studies is an important limitation to consider. For instance, the median follow-up used for eGFR calculation varied from 3 mo in the study by Anderson et al. [5] to 19 mo by Sharma et al. [21]. The best method for estimating GFR would have been renal scintigraphy, as used in the CLOCK trial [4]. Lastly, the PSM rate was used as a proxy for oncologic outcomes in some studies.

In conclusion, our aim was to provide an updated review on the topic and we selected randomized or at least matched studies with a focus on RAPN. Despite their limitations, the published randomized trials provide answer to the research question. It is unlikely that there will be further randomized trials on this topic: the authors of the available RCTs have lived the frustration of the impossibility of an ideal randomized trial about this surgical topic, given the high potential for deviations from the randomization protocol.

On the basis of results from these trials and our pooled analyses, an off-clamp or on-clamp approach to the renal hilum may remain a choice that each surgeon makes according to their own preference: avoiding clamping of the renal artery does not provide any clinically relevant benefit in terms of renal function, but it does not add any additional harm in terms of bleeding, complications, and PSMs.

## 4 Conclusions

In this pooled analysis of studies comparing off-clamp versus on-clamp RAPN, we observed no difference between the two groups in terms of the percentage decline in eGFR at the last available follow-up. Furthermore, there were no significant differences in operative time (on sensitivity analysis), complication rates, need for blood transfusion, EBL, or conversion to open or radical surgery between the groups. The PSM rate was lower in the off-clamp group. Selection bias and heterogeneity for the studies remain as limitations of our study.

  ***Author contributions:*** Gopal Sharma had full access to all the data in the study and takes responsibility for the integrity of the data and the accuracy of the data analysis.

  *Study concept and design*: Shrivastava, Sharma, Ahluwalia, Gautam.

*Acquisition of data*: Shrivastava, Sharma, Ahluwalia, Gautam.

*Analysis and interpretation of data*: Shrivastava, Sharma, Ahluwalia, Gautam, Bertolo, Campi.

*Drafting of the manuscript*: Shrivastava, Sharma, Ahluwalia, Gautam, Bertolo, Campi.

*Critical revision of the manuscript for important intellectual content*: Shrivastava, Sharma, Ahluwalia, Gautam, Bertolo, Campi, Roussel, Pavan, Marchioni, Amparore, Erdem, Marandino.

*Statistical analysis*: Sharma.

*Obtaining funding*: None.

*Administrative, technical, or material support*: Sharma, Campi.

*Supervision*: Sharma, Campi.

*Other*: None.

  ***Financial disclosures:*** Gopal Sharma certifies that all conflicts of interest, including specific financial interests and relationships and affiliations relevant to the subject matter or materials discussed in the manuscript (eg, employment/affiliation, grants or funding, consultancies, honoraria, stock ownership or options, expert testimony, royalties, or patents filed, received, or pending), are the following: Puneet Ahluwalia and Gagan Gautam are proctors for Intuitive Surgical. The remaining authors have nothing to disclose.

  ***Funding/Support and role of the sponsor:*** None.

  ***Data sharing:*** The data can be provided on request to genuine authors.
