# Supplementary material for: Off-clamp Versus On-clamp Robot-assisted Partial Nephrectomy: A Systematic Review and Quantitative Synthesis by the European Association of Urology Young Academic Urologists Renal Cancer Study Group
Source: Eur Urol Open Sci. 2023 Oct 28;58:10–8. doi: 10.1016/j.euros.2023.10.001 (PMC10630115; doi:10.1016/j.euros.2023.10.001)
Supplement: Supplementary data 1 [file mmc1.docx]

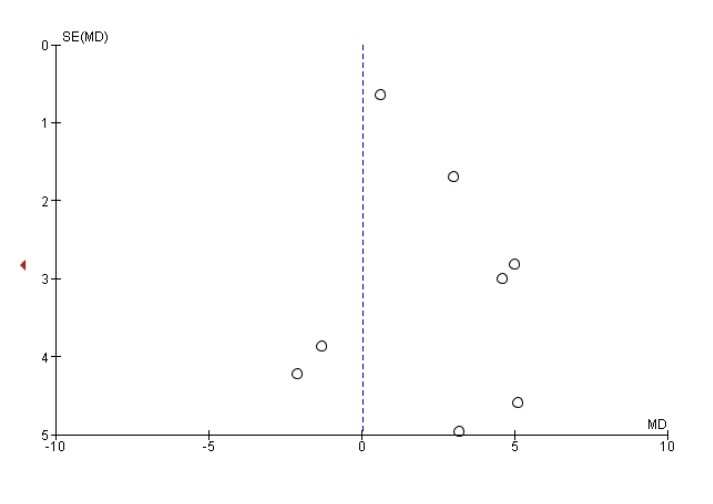


Supplementary Fig. 1 – Funnel plot for the primary outcome of the included studies.

**Supplementary Table 1: Search strategy for PubMed.**

| Query | Search Details | Results |
| --- | --- | --- |
| ((robot assisted partial nephrectomy) AND ((on-clamp) OR (on clamp))) AND (((off-clamp) OR (off clamp)) OR (clampless)) | ("robot"[All Fields] OR "robot s"[All Fields] OR "robotically"[All Fields] OR "robotics"[MeSH Terms] OR "robotics"[All Fields] OR "robotic"[All Fields] OR "robotization"[All Fields] OR "robotized"[All Fields] OR "robots"[All Fields]) AND ("assistances"[All Fields] OR "assistant s"[All Fields] OR "assistants"[All Fields] OR "assisted"[All Fields] OR "assisting"[All Fields] OR "assistive"[All Fields] OR "dental assistants"[MeSH Terms] OR ("dental"[All Fields] AND "assistants"[All Fields]) OR "dental assistants"[All Fields] OR "assistant"[All Fields] OR "helping behavior"[MeSH Terms] OR ("helping"[All Fields] AND "behavior"[All Fields]) OR "helping behavior"[All Fields] OR "assist"[All Fields] OR "assistance"[All Fields] OR "assists"[All Fields]) AND ("partial"[All Fields] OR "partials"[All Fields]) AND ("nephrectomy"[MeSH Terms] OR "nephrectomy"[All Fields] OR "nephrectomies"[All Fields]) AND ("on-clamp"[All Fields] OR ("clamp"[All Fields] OR "clamped"[All Fields] OR "clamps"[All Fields] OR "constriction"[MeSH Terms] OR "constriction"[All Fields] OR "clamping"[All Fields] OR "clampings"[All Fields])) AND ("off-clamp"[All Fields] OR ("off"[All Fields] AND ("clamp"[All Fields] OR "clamped"[All Fields] OR "clamps"[All Fields] OR "constriction"[MeSH Terms] OR "constriction"[All Fields] OR "clamping"[All Fields] OR "clampings"[All Fields])) OR "clampless"[All Fields]) | 63 |
| (on-clamp) OR (on clamp) | "on-clamp"[All Fields] OR "clamp"[All Fields] OR "clamped"[All Fields] OR "clamps"[All Fields] OR "constriction"[MeSH Terms] OR "constriction"[All Fields] OR "clamping"[All Fields] OR "clampings"[All Fields] | 162,214 |
| on clamp | "clamp"[All Fields] OR "clamped"[All Fields] OR "clamps"[All Fields] OR "constriction"[MeSH Terms] OR "constriction"[All Fields] OR "clamping"[All Fields] OR "clampings"[All Fields] | 162,214 |
| ((off-clamp) OR (off clamp)) OR (clampless) | "off-clamp"[All Fields] OR ("off"[All Fields] AND ("clamp"[All Fields] OR "clamped"[All Fields] OR "clamps"[All Fields] OR "constriction"[MeSH Terms] OR "constriction"[All Fields] OR "clamping"[All Fields] OR "clampings"[All Fields])) OR "clampless"[All Fields] | 2,512 |
| on-clamp | "on-clamp"[All Fields] | 88 |
| clampless | "clampless"[All Fields] | 156 |
| off clamp | "off"[All Fields] AND ("clamp"[All Fields] OR "clamped"[All Fields] OR "clamps"[All Fields] OR "constriction"[MeSH Terms] OR "constriction"[All Fields] OR "clamping"[All Fields] OR "clampings"[All Fields]) | 2,395 |
| off-clamp | "off-clamp"[All Fields] | 149 |
| robot assisted partial nephrectomy | ("robot"[All Fields] OR "robot s"[All Fields] OR "robotically"[All Fields] OR "robotics"[MeSH Terms] OR "robotics"[All Fields] OR "robotic"[All Fields] OR "robotization"[All Fields] OR "robotized"[All Fields] OR "robots"[All Fields]) AND ("assistances"[All Fields] OR "assistant s"[All Fields] OR "assistants"[All Fields] OR "assisted"[All Fields] OR "assisting"[All Fields] OR "assistive"[All Fields] OR "dental assistants"[MeSH Terms] OR ("dental"[All Fields] AND "assistants"[All Fields]) OR "dental assistants"[All Fields] OR "assistant"[All Fields] OR "helping behavior"[MeSH Terms] OR ("helping"[All Fields] AND "behavior"[All Fields]) OR "helping behavior"[All Fields] OR "assist"[All Fields] OR "assistance"[All Fields] OR "assists"[All Fields]) AND ("partial"[All Fields] OR "partials"[All Fields]) AND ("nephrectomy"[MeSH Terms] OR "nephrectomy"[All Fields] OR "nephrectomies"[All Fields]) | 1,338 |
